# Probiotic lactobacilli in formulas and hygiene products for the health of the urogenital tract

**DOI:** 10.1002/prp2.787

**Published:** 2021-10-05

**Authors:** María Elena Fátima Nader‐Macías, Priscilla Romina De Gregorio, Jessica Alejandra Silva

**Affiliations:** ^1^ Centro de Referencia para Lactobacilos‐Consejo Nacional de Investigaciones Científicas y Técnicas de Argentina (CERELA‐CONICET) San Miguel de Tucumán Argentina

**Keywords:** clinical trials, female hygienic products, female urogenital tract, live biotherapeutic products, probiotics, urogenital tract infections

## Abstract

Lactobacilli are the predominant microorganisms of the healthy human vagina. A novel alternative for the prevention and treatment of female urogenital tract infections (UGTI) is the inclusion of these microorganisms as active pharmaceutical ingredients in probiotic formulas, and more recently in female hygienic products. Probiotics are defined as “live microorganisms that, when administered in adequate amounts, confer a health benefit on the host.” A list of requirements must be considered during the development of probiotic product/formula for the female urogenital tract (UGT). This review aims to resume the requirements, probiotic characteristics, and clinical trial applied to determine the effect of probiotic and potentially probiotic strains on different woman’s physiological and pathological conditions, and in preterm birth prevention. A revision of female hygienic products available in the world market is included, together with novel studies applying nanotechnology for *Lactobacillus* incorporation in hygienic products. Further studies and well‐designed clinical trials are urgently required to complement the current knowledge and applications of probiotics in the female UGT. The use of probiotic formulas and products will improve and restore the ecological equilibrium of the UGT microbiome to prevent and treat UGTI in women under different conditions.

**Abbreviations**
AV: aerobic vaginitis
BV: bacterial vaginosis
DNA: deoxyribonucleic acid
GBS: Group B *Streptococcus*
GDM: gestational diabetes mellitus
GRAS: generally regarded as safe
HIV: human immunodeficiency virus
HM: human microbiome
HPV: human papilloma virus
HR‐HPV: high risk human papilloma virus
HSV‐2: Herpes simplex virus type 2
IM: intestinal microbiota
LBP: live biotherapeutic products
LN: Lacto Naturel
NIH: national institutes of health
PPROM: preterm premature rupture of membranes
QPS: qualified presumption of safety
R‐VVC: recurrent‐VVC
SR: slow‐release
TV: trichomoniasis
UD: undetermined
UGT: urogenital tract
UGTI: urogenital tract infections
UTI: urinary tract infection
VM: vaginal microbiota
VVC: vulvovaginal candidiasis

## 1 HEALTHY VAGINAL MICROBIOTA—LACTOBACILLUS PREDOMINANCE: FUNCTIONS

The human body and their mucosa are lately considered as highly active ecosystems, where microorganisms play a very diverse list of functions and contribute to host nutrition, overall development, defenses, and immune system, response to pathogens, and mucosal cell differentiation and proliferation. The related research and the knowledge of these active communities and their gene contents have been referred collectively as the human microbiome (HM), supported mainly by an NIH‐funded project consortium.^1^ The Human Microbiome Project and the European MetaHIT consortium initiated almost two decades ago, aimed at detailed characterization of the structure and the composition of the microbiota from various body areas.^1^ It is interesting to remark that microbial numbers within an individual are estimated higher than the human cell number by an order of magnitude. In such a way that the HM is a complex system of many microbial communities, deeply described in terms of composition, diversity, and dynamics, known thanks to the use and update of massively parallel sequencing and other high throughput approaches available, indicating the long list of eukaryotes, archaea, bacteria, and viruses detected. These molecular techniques include Sanger sequencing of 16S rRNA of bacterial colonies, terminal restriction fragment length polymorphism of 16S rRNA, qPCR, and next‐generation sequencing that have modified the concept of the microbiome versatilities and more specifically *Lactobacillus* identification.^2^, ^3^, ^4^, ^5^, ^6^, ^7^, ^8^, ^9^, ^10^, ^11^, ^12^, ^13^

Then, the majority of the indigenous microbiota exists in a mutually beneficial relationship with the host, while few are opportunistic pathogens.^14^ An important body site providing a habitat for the development of structured microbial communities is the vaginal tract, which is broadly colonized by microorganisms known as the vaginal microbiota (VM). Unique conditions of the vagina are characterized by a few microbial species, being the vaginal microbiome a specific compartment of the HM.^15^ The predominance of lactobacilli was described deeply in the vaginal tract in the NIH Project, and this concept agrees with the first proclaimed by Doderlein in the early 1900.^16^ The most frequently isolated species are *Lactobacillus crispatus*, *L*. *gasseri*, *L*. *jensenii*, and *L*. *iners*. Their relative dominance was studied by Ravel et al.^8^ in different races and ethnic groups. They evidenced the prevalence of *L*. *crispatus* (group I), *L*. *gasseri* (group II), *L*. *iners* (III), and *L*. *jensenii* (V) in 26.2%, 6.3%, 34.1%, and 5.3% of the women sampled, respectively. A large heterogeneous group (IV) was presented in 27% of the women with a higher proportion of strictly anaerobic bacteria (*Prevotella*, *Dialister*, *Atopobium*, *Gardnerella*, *Megasphaera*, *Peptoniphilus*, *Sneathia*, *Eggerthella*, *Aerococcus*, *Finegoldia*, and *Mobiluncus*). These results indicate that a potential key ecological function, the production of lactic acid, seems to be conserved in all communities, and support at the same time the application of lactic acid producer and immunomodulatory‐probiotic bacteria, and are concordant with α diversity studies published later.^17^, ^18^, ^19^ Ma and Li^20^ have published recently a review on the association between vaginal community state types and species specificity index reclassifying in five groups.

The second phase of the NIH‐Human Microbiome Project was the Integrative Human Microbiome Project^21^ designed to explore host–microbiome interplay, including immunity, metabolism, and dynamic molecular activity to gain a more holistic view of host–microbe interactions over time, in a way to address the relationships between host and microbiome mechanistically. The enormous importance of vaginal microbiome supported its inclusion as one of the systems proposed to be studied in this second phase: pregnancy and preterm birth, to go further in the dynamics of human health and disease‐related with known microbiome interactions. In such a way that the concept of *reproductive microbiome* has been conceived, joining the microbiome of the vaginal tract, placenta and milk/mammary gland, and the direct relationship with fetal development, birth, and newborn features.^22^ In addition, the VM of the mother plays an essential role in the initial colonization of newborn babies and therefore the development of a healthy gastrointestinal and skin microbiota. The maternal microbiota exerts an indirect effect on the fetus via maternal factors, such as maternal immune responses or microbial metabolites that cross the placenta,^23^, ^24^ or other indirect factors mediating epigenetic programming in the fetus (diet, stress, or neuroendocrine exposure). Kaminska and Gajecka^25^ discuss the influence of human VM, not only bacteria but also viruses and fungi that constitute important components of the reproductive tract microbiome. The impact of the maternal microbiome on fetal development, and the establishment of neonatal microbiomes, including the placenta microbiome, and the hematogenous source of intrauterine infection on the health status of women were analyzed. On the other side, some evidence indicates that infertile patients harbor a different reproductive tract microbiome compared with healthy and fertile women.^26^

Fertilization occurs in the uterus, an immune‐protected organ, considered a sterile site maintained by the cervical plug for centuries. But the microbial communities in the endometrial cavity and its implications in reproductive health and disease, particularly chronic endometritis was published.^27^ Even though bacterial DNA in blood in placenta was demonstrated, mechanisms and functions of transplacental trafficking of free nucleic acids are still in discussion. Then, microbes interact with the host cells along the female reproductive tract, generating the physical, chemical, and biological environment that embryo will encounter during the peri‐implementation period and throughout pregnancy. Later, birth represents the first major exposure to a complex microbiota, because the birth canal is always adjacent to the rectum, providing an efficient mechanism for intergenerational transmission of both vaginal and gut microbes.^28^ The baby swallows these microorganisms, supported by DNA and live bacteria in the meconium.^29^ The maternal gut microbes immediately start to colonize the newborn's own gut, engaging in a kind of conversation with developing immune cells. In this way, the very early microbiome prepares the immune system for healthy functioning later in life. When a baby is born by cesarean section, the gut is seeded with different microbes not those from the mother's gut and vagina, but from her skin and breast milk, the nurse's hands, and other sources. These early differences might have implications that last a lifetime and provides differential colonization and diversity of microorganisms in the intestinal microbiome in C‐sections or vaginal born babies later in their lives.^30^, ^31^

On the other hand, the urinary tract, previously considered a sterile body niche, has emerged as the host of an array of bacteria in healthy individuals, revolutionizing the urology research field.^32^ Specific bacterial communities have found in the healthy urinary tract, which can change in a wide variety of urologic disorders. Then, it is also of main importance to resolve the modulation of the microbiome to improve urinary tract health.

## 2 FACTORS AFFECTING THE VM EQUILIBRIUM

The equilibrated/healthy ecological systems of the reproductive microbiota can be affected by a long list of intrinsic or extrinsic factors. *Intrinsic factors or host factors* include race, physiologic (hormonal changes, menstruation, pregnancy), immune system imbalances, maturation, and the relationship with genetic susceptibility, cancer, and the phages isolated in the tract.

The VM of reproductive‐age women was separated according to the *ethnic groups* (White, Black, Hispanic, and Asian) in five clusters, showing that the proportions of each community group varied among them.^8^
Women’s life is characterized by *continuous physiological changes*, from their birth through the reproductive age to menopause, and during all these phases the vaginal epithelium radically changes, and then the VM.^33^ Monthly ovulation, with high estrogenic levels, lead to vaginal tract, particularly acid, optimum for the lactobacilli growth, producing lactic acid.^33^, ^34^, ^35^ During the menstrual period there is an increment of pH in the area, and high availability of nutrients derived from menstrual bleeding for microbial growth. This period usually causes disturbance and discomfort, with lower number of lactobacilli, consequent undesirable microorganisms, increased infection rates, and recurrences.^8^, ^36^, ^37^ In menopause, the estrogens are no longer present and glycogen level decreases, leading to a decrease in lactobacilli.^38^
The human *immune system* restricts microbiota to their natural niches.^28^ There is growing evidence that the innate immune system‐antimicrobial peptides and repertoire of pattern recognition receptors, evolved in response to the need for controlling the epithelium‐colonizing microbiota.^39^ The human vagina consists of multiple levels of protection in innate and adaptive immunity compartmentalize into various components.^40^, ^41^ On this subject, genetic variations, such as single nucleotide polymorphisms, in different genes coding components of immune system have been shown to modulate individual´s susceptibility to acquire urogenital tract infections (UGTI).^42^
*Bacteriophages* are abundant members of the urogenital tract (UGT), most often persisting through the lysogenic life cycle as prophages integrated within the genomes of their bacterial hosts. Numerous prophages in vaginal lactobacilli were related as one of the factors affecting their depletion in vagina.^43^, ^44^

### 2.1 Extrinsic factors

A long list and variety of factors affect the microbiota equilibrium, as the addition of vitamins and folic acid to diet,^45^, ^46^, ^47^ oral anticontraceptives,^48^ sexual behaviors,^49^ hygiene habits,^50^ stress situations,^51^ sexually transmitted diseases,^52^ antibiotics,^53^ or immunosuppressor therapies,^54^ between others.

## 3 UGTI AND FREQUENTLY APPLIED THERAPY

Female vaginal ecosystem thought to have been shaped over the years by co‐evolutionary processes occurring between the particular microbial partners and the human host. Residing at the port of entry of pathogens, the vaginal *Lactobacillus* species can create a barrier against pathogen invasion since the main products of their metabolism secreted in the cervicovaginal fluid can play an important role in inhibiting invaders. Therefore, a *Lactobacillus*‐dominated microbiota appears to be a good biomarker for a healthy vaginal ecosystem.^55^ Disruptions in vaginal association with the microbiomes lead to the change in the vaginal environment, which enhanced the risk of acquiring diseases. The disturbed population promotes a loss of beneficial bacteria due to the list of exogenous/endogenous factors listed above. Thus, vaginal tract can be infected by diverse pathogens resulting in diseases such as bacterial vaginosis (BV), vulvovaginal candidiasis (VVC), trichomoniasis (TV), urinary tract infections (UTI), aerobic vaginitis (AV), and sexually transmitted diseases as those caused by *Chlamydia trachomatis*, *Neisseria gonorrhoeae*, *Treponema pallidum*.^14^ The main viral sexually transmitted diseases are human papilloma virus (HPV), human immunodeficiency virus, and herpes simplex virus type 2 (HSV‐2). Each pathogen has its unique infection kinetics, pathology, and host evasion mechanisms. In a similar way, the genital mucosal immunity reacts differently toward each of them.

The urinary tract has emerged as the host of an array of bacteria in healthy individuals. There is a microbiome associated with the healthy urinary tract that can change in certain urological disorders.^32^, ^56^ The vagina is a key anatomical site in the pathogenesis of UTI in women, serving as a potential reservoir for infecting bacteria, and also the organ where the ascendant colonization from rectum begins.^57^ UTIs in females usually start as vaginal infections and ascend to the urethra and bladder.^58^ Women are affected with an estimated UTI lifetime risk of 60%, and 50%‐fold predominance compared with adult men^59^ increasing with age.^60^ Single episodes are very common, being *Escherichia coli* the most common cause of UTI^57^, ^61^ or different Gram‐positive bacteria.^62^ Recurrent UTI occurring in up to one‐third of women after the first episode,^63^ showed to be related to a decreased vaginal lactobacilli.^64^ Rates of UTI begin to rise at the climateric, and recurrences are one of the features of menopause.^65^ Vaginal tract is then a site at which probiotic interventions may decrease the risk of UTI.^57^, ^58^, ^66^

A wide diversity of antimicrobial compounds is applied to treat UGTI by vaginal or oral administration, including antibacterial, antifungal, antiparasitic, and antiviral agents. The antibiotic use decreases the vaginal *Lactobacillus* number, promotes resistance, and sometimes infection recurrences. In such a way that the resistance to antimicrobials can be transmitted either transversally or horizontally to other members of the microbiome, generating multi‐resistant microorganisms. The “*resistome*” concept has emerged, representing the collection and variety of genes that confer antimicrobial resistance (biocides, heavy metals, plasmids) in different microbiomes, spread over multiple areas,^67^ analyzed lately by targeted metagenomics techniques.^68^

For this reason, and supported by different concerns related to public health and the prevention of infections, the urgent requirement of adequate alternatives emerged in the last two decades.

## 4 PROBIOTIC AS PROPOSAL THERAPIES. STRAIN, AND HOST SPECIFICITY

One of the therapies applied for UGTI are probiotics. The definition was submitted to many discussions by government and scientific organizations during the last 30 years, and at the end is *live microorganisms that, when administered in adequate amounts,* *confer a physiological health benefit on the host*.^69^, ^70^ However, more recently, new definitions have complemented the probiotic area. The term “paraprobiotics” means dead or inactive cells of probiotics that have shown a significant effect on human health^71^, ^72^, ^73^, ^74^; while the term “postbiotics” is used to describe healthful metabolites of probiotics also showing effect on the host.^75^, ^76^, ^77^, ^78^ Lately, Zendeboodi et al.^79^ proposed three main classes of probiotic products, including “true probiotic” as viable and active probiotic cell, “pseudo‐probiotic” as viable and inactive cells in vegetative or spore forms, and “ghost probiotic” as dead/nonviable cell, either intact or ruptured.

Probiotics have been widely studied and applied in the field of food and food supplements, with no particular negative effect.^80^, ^81^, ^82^ In the last few years, the pharmaceutical industry showed a growing interest in new formulations containing beneficial microorganisms, in many cases specific to the host.^83^, ^84^ However, the increased interest in the clinical application of probiotics requires specific attention by their administration to non‐healthy population. More recently, the Food and Drug Administration has defined a new “live biotherapeutic products” (LBP) category. Then, the documents and demonstration of quality, safety, and efficacy of new products, including LBP, are framed by the characteristics and risks of the aimed population, as well as those of the strains and product components, in such a way that the global benefit–risk ratio can be assessed regarding their intended use.^85^

Not all the *Lactobacillus* strains can be considered as probiotics because the beneficial effect must be evidenced in the specific host as stated. The strains included in different products/formulas must be submitted to a long list of protocols and trials to be named as probiotics. Also, a body of different criteria applied, reviewed by different authors, to be considered as probiotic strains.^69^, ^86^ The range of functional genomics to identify genes and gene products that govern the distinctive phenotypes and health associations of probiotic strains was reviewed, recognizing the strain specificity of probiotic effects.^87^ This strain specificity and the disease specificity of probiotic efficacy, and the need to take these two factors: both specific strains and type of disease, when recommending the appropriate probiotic effect for each patient is also recommended.^88^, ^89^ One of the first considerations is the *host specificity*, which means that the strains should be isolated from the same host where will be applied. Even though some strains are used historically as probiotic or included in foods, the host specificity indicates a higher possibility to be adapted to the host conditions. Referred to the *vaginal* *tract or mucosal specificity*, Yildirim et al.^90^ have shown that all primates exhibited host‐specific VM and that humans were distinct from other primates in both microbiome composition and diversity. Mendes‐Soares et al.^91^ demonstrated that genomes of vaginal species were significantly smaller and had significantly lower GC content than those of non‐vaginal species.

More specifically, Van Der Veer et al.^92^ have found that *L*. *crispatus* from dysbiotic women have a more prevalent novel glycosyltransferase gene, while Pan et al.^89^ evidenced strain‐specific variations and phenotypic differences associated with the isolation source, either urogenital or gastrointestinal tracts.

## 5 REQUIREMENTS FOR PROBIOTICS FORMULAS/PRODUCTS

**FIGURE 1:**
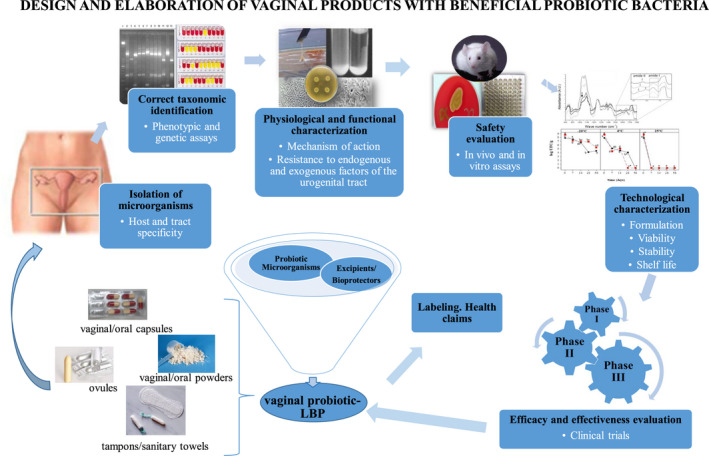
Design and elaboration of vaginal products with beneficial probiotic bacteria (modified from reference [86]). LBP, live biotherapeutic products

In the design of probiotic formulas/products, different sets of criteria and protocols must be applied according to the requirements and exigencies of regulatory organisms, described in different revisions, added to the host and tract specificity described above.^86^, ^93^, ^94^ They include: (a) place of isolation, (b) correct phenotypic and genetic identification of the strains, (c) functional characterization and mechanisms of action, (d) in vitro and in vivo safety evaluation, (e) technological characterization: production, formulation, and shelf life, (f) efficacy and effectiveness in clinical trials, and (g) label and health claims; and they are synthesized in Figure 1.

Most of the probiotic lactobacilli are considered as Generally Regarded as Safe and some specific species included in the Qualified Presumption of Safety (QPS) classification for inclusion in food,^95^ but their effectiveness and safety characteristics are strain‐specific and cannot be generalized. Then, no new strain should be assumed of sharing the same documented safety history with preexisting ones.^96^ Different efficacy and safety assessment protocols were recommended by the experts for a putative probiotic candidate, before confirmation and acceptance for public consumption. In order to guarantee the probiotic safety, each strain must be assayed in some aspects: antibiotic‐resistance patterns; assessment of certain metabolic activities; toxin production; determination of hemolytic activity; side‐effects in experimental animal models; phase I trials; epidemiological surveillance of adverse incidents in consumers, between other.^69^ Refered to the potential dissemination of the resistance mediated by genetic mechanisms, such as horizontal gene transfer where plasmids, transposons and integrons may be involved,^97^, ^98^, ^99^, ^100^, ^101^ is essential to evaluate the susceptibility patterns of potentially probiotic bacteria.^102^ Microbial resistance to clinically relevant antibiotics must be absent as part of the QPS assessment of bacteria deliberately introduced into the food chain.^103^ Also it is important to screen the enzymes acting as potential virulence factors, as hydrolytic enzymes (hemolysin, lecithinase, gelatinase, etc.) able to produce damage to the host.^104^, ^105^, ^106^, ^107^ The temporal persistence and colonization or permanence capability of beneficial microorganisms, and their cellular and molecular effects on the integrity of host mucosa and immune system must also be assayed.^108^, ^109^, ^110^

The formulas for the UGT are designed for oral or local administration. The oral delivery is supported by the microbial ability to survive through gastrointestinal tract, and to ascend to vagina after their excretion from the rectum, in ascendant colonization.^111^, ^112^, ^113^ Whereas vaginal application allows direct and targeted colonization of bacteria for the restoration of unbalanced urogenital microbiota.^114^, ^115^, ^116^, ^117^, ^118^ The vaginal drug delivery systems include a wide variety of pharmaceutical forms such as liquid, semi‐solid (gels, creams, ointments), and solid systems (tablets, vaginal suppositories, rings, films, tampons). The viable bacteria are incorporated frequently as powder, being lyophilization the gold standard technique for microbial preservation and stability during the storage for different time periods.^119^, ^120^, ^121^, ^122^ The selection of the appropriate dosage form depends on the physicochemical features of the delivered drug or formula, the target for them and women´s acceptance.^123^

## 6 EFFECTS OF PROBIOTIC OR POTENTIAL PROBIOTIC MICROORGANISMS ON THE FEMALE UGT

**FIGURE 2:**
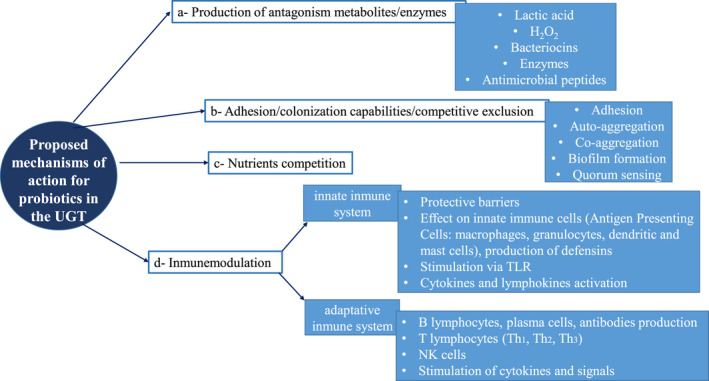
Proposed mechanisms of action for probiotics in the UGT (adapted from different bibliographic references). TLR, toll‐like receptor; UGT, urogenital tract

Pharmacological aspects of probiotics are more complex than those of conventional drugs, since their effect in the host cells‐mucosa‐organs and whole organism is very complex and difficult to evaluate, because they act by different mechanisms, either individually or in some cases synergistically. Several publications describe how vaginal lactobacilli can exert their function through different mechanisms of actions, which include: (a) production of antimicrobial substances (organic acids, hydrogen peroxide, bacteriocins, biosurfactants) or enzymes (e.g. arginine deaminase); (b) adhesion to epithelial cells, components of mucus or extracellular matrix‐colonization‐permanence; (c) autoaggregation and coaggregation; biofilm formation on vaginal mucosa; (d) competitive exclusion; (e) competition for nutrients; and (f) modulation of the immune system.^86^, ^94^, ^124^, ^125^, ^126^, ^127^, ^128^, ^129^, ^130^, ^131^, ^132^ The proposed mechanisms of action of probiotics in the UGT are shown in Figure 2.

A very important subject to be considered in the development of probiotic formulas for the UGT is the effect of the bacteria as bioactive principle, whether for preventive or therapeutic purposes, which is supported by the reestablishment of the ecological microbial equilibrium of the tract. One of the main requirements of probiotic products is the viable cell number, between 10^7^ and 10^9^ colony forming units per formulation dose.^70^, ^94^, ^133^, ^134^ Since the effectiveness of probiotic formulas depends on these number of viable cells, most of the pharmacokinetic studies have described the survival capability of probiotics in the target organ and its capability to maintain their numbers and colonize to generate the probiotic effects. The survival of microorganisms depends on their resistance to the conditions of the host tract and to the technological processes and their maintenance as marketed products. The fate of probiotics (i.e., their survival, or movement) in the gastrointestinal tract (or in experimental animal models), their effect in specific target organs, or stimulation of specific cell‐populations, mainly in the gut were demonstrated. Scientific publications evidenced that oral probiotics increase intestinal antimicrobial activity and Paneth cells in order to strength the epithelial barrier against pathogens in mice, proposing a different mechanism by which probiotics protect the host mainly against infectious diseases. The whole bacteria can not enter the intestinal cells, while only the degradation products of bacteria are able to take contact with the immune cells.^135^, ^136^ A recent study have shown the probiotic properties of two vaginal lactobacilli (*L*. *fermentum* MG901 and *L*. *plantarum* MG989), the adhesion to HT‐29 cell and the inhibition of *E*. *coli* and *Candida albicans* adherence to these cells. The probiotic bacteria persisted up to 6 days in the feces of mice after the oral administration. The authors suggested the vaginal strains could be used as oral and vaginal probiotic helping to in vivo clear VVC.^137^

Referred to oral probiotics for women, supported by the ascending colonization hypothesis that promotes the permanence and colonization of beneficial bacteria in the vagina after excretion through the rectum,^111^, ^112^, ^113^ the capability of microorganisms to colonize the vagina was studied. De Vrese et al.^138^ showed that oral intake of four *Lactobacillus* strains improves the microbial pattern in vaginal dysbiosis. In a similar way, Vladareanu et al.^139^ demonstrated that oral probiotic increased the vaginal colonization of lactobacilli via cross‐contamination from the gastrointestinal tract to vagina, indicating an improvement of vaginal conditions, suggesting then the successfully prevention of VVC episodes.

On the other side, the effect, safety, and mechanisms of action of lactobacilli as probiotic products for local administration, such as vaginal capsules, suppositories, and tablets, were demonstrated in different publications. Verdenelli et al.^140^ registered the normalization and maintenance of pH and Nugent score and the increase of lactobacilli total number after 7 days of probiotic suppositories treatment in women. The authors highlighted that local administration promotes a quick local action, driven by the activity of probiotic bacteria that adhere and colonize the vaginal epithelium. The colonization of vaginal epithelium was shown by molecular typing test, suggesting the restoration and maintenance of VM. Cohen et al.^141^ published a randomized, double‐blind, placebo‐controlled, phase 2b trial to evaluate Lactin‐V (*L*. CTV‐05) after the treatment with vaginal metronidazole, resulting in a significantly lower incidence of BV recurrence.

The use of probiotics in the obstetrical and gynecological field has increased in the last years. Up to date, several clinical studies have evaluated the effects of probiotic or potential probiotic microorganisms, administered both orally and vaginally, on the prevention and treatment of UGTI in women under different physiological conditions: fertile and non‐pregnant, pregnant, and post‐menopausal, as well as also in the prevention of preterm birth (Table 1). In the present review, most of the available clinical trials published between 2015 and 2020 were included, because previous trials were analyzed before.^86^, ^142^

**TABLE 1:** Effect of probiotic or potential probiotic microorganisms on the female urogenital tract

Microorganisms	Isolation site	Pharmaceutical form or product	Clinical target^a^	Type of study and participant type and number	Results	References
*Lactobacillus crispatus* CTV‐05	Healthy human vagina	Vaginal powder (Lactin‐V, Osel)	Subsequent to BV therapy	Multicenter randomized double‐blind placebo‐controlled phase 2b trial. 228 BV women	Probiotic (11 weeks) after metronidazole produced lower incidence BV recurrence	141
*L*. *plantarum* P17630	Healthy human vagina	Oral capsule (Proge Farm S.r.l.)	VM‐improvement	Randomized double‐blind placebo‐controlled study. 93 R‐VVC history women	Probiotic [3 treatment cycles (15 days/cycle) separated by 15‐day wash‐out intervals] improved *Lactobacillus* colonization and clinical sign (redness/swelling/discharge)	139
*L*. *rhamnosus* Lcr35^®^	Human feces	Vaginal capsule or slow‐release (SR) vaginal tablet (Gynophilus^®^)	VM‐improvement	Comparative phase I randomized open‐label pilot clinical trial. 33 healthy women	Capsules (daily) or SR‐tablets (every 3, 4, or 5 days for 21 days) did not produce adverse effects, favored *Lactobacillus* spp. growth, reduced non‐*Lactobacillus* spp. colonization	143
		Vaginal capsule	Adjunct to therapy of TV in BV‐presence	Randomized placebo‐controlled double‐blind study. 90 women with TV in BV presence	Probiotic (1 capsule twice/day/7 days) increased TV and BV cure, decreased vaginal inflammation and pH, increased redox potential	144
*L*. *rhamnosus* BMX 54	UD	Vaginal tablet (NORMOGIN^®^)	VM‐restoration	Randomized trial. 117 HPV+BV or VVC‐women receiving standard antimicrobial treatment	Probiotic (6 months) resolved HPV‐related cytological anomalies twice higher than probiotics during 3 months, induced high total HPV clearance	145
*L*. *rhamnosus* HN001	Yoghurt	Oral capsule (Fonterra‐Cooperative)	Improvement‐maternal health‐pregnancy (prevention maternal GDM) and postpartum (depression‐anxiety), prevention infant eczema‐allergy	Two‐center randomized double‐blind placebo‐controlled trial. 423 pregnant women at 14–16 weeks gestation with personal or partner history of atopic disease and expecting infants at high risk of allergic disease	Probiotic (until delivery and then until 6 months post‐partum, if breastfeeding), reduced GDM prevalence, decreased depression and anxiety scores in the postpartum period, did not reduce infant eczema	146, 147, 148, 149
*L*. *salivarius* CECT 9145	Healthy human vagina	Freeze‐dried	GBS‐vaginal and rectal colonization‐reduction	Prospective pilot clinical trial. 57 pregnant women	Lactobacilli (one/day from week 26/38 pregnancy) reduced rectal and vaginal GBS colonization	150
*L*. *acidophilus* La‐14, *L*. *rhamnosus* HN001	Human feces and yoghurt, respectively	Oral capsule+bovine lactoferrin RCX^™^ (Respecta^®^)	VM‐improvement	Double‐blind randomized placebo‐controlled study. 40 healthy women	Probiotic (twice daily/2 weeks) increased probiotic species in vagina and without adverse effect	151
			Adjunct to BV‐therapy	Double‐blind placebo‐controlled‐randomized clinical trial. 48 BV women	Probiotic+lactoferrin (2 capsules/day/5 days followed by 1 capsule/day/10 days/month during 6 months) reduced vaginal discharge, itching, nugent score and recurrence rate	37
			Adjunct to R‐VVC‐therapy	Double‐blinded placebo‐prospective randomized clinical trial. 48 *Candida albicans*‐positive women	Probiotic+lactoferrin (2 capsules/day/5 days followed by 1 capsule/day/10 days/month during 6 months) reduced itching and discharge at 3 and 6 months, and R‐VVC	152
*L*. *rhamnosus* IMC 501, *L*. *paracasei* IMC 502	Elderly human faeces Human feces	Vaginal suppository (SYNBIO^®^)	VM‐restoration	Single‐arm open‐label controlled towards the baseline (pre–post) study. 35 apparently healthy women	Probiotic for 7 days did not produce adverse effects, reduced Nugent score, increased *Lactobacillus* level, did not modify pH vaginal	140
*L*. *rhamnosus* GR‐1, *L*. *reuteri* RC‐14	Healthy human urethra and vagina, respectively	Oral capsule (U‐relax^®^)	HR‐HPV‐clearance	Randomized double‐blinded placebo‐controlled trial. 121 HR‐HPV women	Probiotic (1 capsule/day until negative HR‐HPV testing) did not influence HR‐HPV clearance, decreased rates of mildly abnormal and unsatisfactory cervical smears	153
		Oral capsule (Chr. Hansen)	VM‐modulation	Pilot randomized blinded placebo‐controlled trial. 38 pregnant women of gestational age less than 36 weeks	Probiotic (1 capsule/day/1 month) without side effects did not modify VM	154
			VM‐maintenance/ restoration	Randomized placebo‐controlled triple‐blind parallel group trial. 320 women with <12 completed pregnancy weeks	Probiotic (1 capsule/day/8 weeks) did not modified VM	155
			VM‐maintenance/restoration	Randomized double‐blind placebo‐controlled trial. 304 women with 9–14 pregnancy weeks	Probiotic (1 capsule/day from recruitment until pregnancy end) did not modified VM	156
			GBS‐vaginal colonization‐reduction	Randomized controlled trial. 99 pregnant women at 35–37 weeks of gestation with vaginal and rectal‐GBS positive	Probiotic (2 capsules before bedtime until delivery) reduced the GBS colonization	157
			GBS‐vaginal colonization‐reduction	Pilot randomized control study. 34 GBS‐positive women at 36 weeks pregnant received standard antenatal care	Probiotic (1 capsule/day/3 weeks or until birth) did not reduce vaginal GBS‐rates	158
*L. fermentum 57A, L. plantarum 57B, L*. *gasseri* 57C	Healthy human vagina	Vaginal capsule (inVag^®^)	VM‐restoration	Multicenter randomized double‐blind placebo‐controlled trial. 160 abnormal VM women	Probiotic (1 capsule/day/7 days) decreased vaginal pH and Nugent score, increased *Lactobacillus* abundance, without adverse effect	159
		Oral capsule (prOVag^®^)	Adjunct to BV/AV‐therapy	Randomized double‐blind placebo controlled trial. 154 of recurrent BV/AV histories and current symptoms women.	Probiotic (2 capsule/day/10 days, during follow‐up, and one capsule/day/10 days perimenstrually), lengthened time to clinical BV/AV symptoms relapse, reduced and maintained low vaginal pH and Nugent score, increased vaginal *Lactobacillus* counts	160
*L*. *rhamnosus* DSM 14870, *L*. *gasseri* DSM 14869	Healthy human vagina	Vaginal capsule (EcoVag^®^)	Adjunct to BV‐therapy	Prospective partially randomized exploratory pilot study. 39 BV women	Probiotic (once/day/30 days then once/week until day 190) colonized vagina, did not improve BV cure rates or alleviate recurrence	161
			Subsequent to BV/R‐VVC‐therapy	Two pilot open‐label clinical trials. 40 Scandinavian BV or VVC‐diagnosed women	Probiotic (5 days) induced a 6‐month BV cure rate of 50%. Probiotic (10 days after each antibiotic treatment followed by weekly administration of capsules for 4 months) induced 6‐ and 12‐month BV‐cure rates of 67%, while the 6‐ and 12‐month VVC‐cure rates of 100% and 89%	162
			Adjunct to antibiotic treatment on perinatal outcome with PPROM	Prospective randomized trial. 115 PPROM between 24 and 34 weeks of gestation women	Probiotic (10 days) increased gestational age at birth, duration of latency period and birth weight. Neonates of probiotic‐group presented lower chance of entering intensive care unit, shorter total hospitalization time, lower need for oxygen administration and mechanical ventilation, and lower length of oxygen administration	163
*L*. *gasseri* LN40, *L*. *rhamnosus* LN113, *L*. *fermentum* LN99	Healthy human vagina	Intimate care ointment (Ellen AB)	VM‐improvement	Double‐blind randomized pilot study. 18 healthy postmenopausal women	Probiotic (10 days) induced lactobacilli persistence in vagina for at least 10 days	164
**Formula A**: *L*. *gasseri* CRL1307, CRL1263, *L*. *reuteri* CRL1324 **Formula B:** *L*. *gasseri* CRL1256, CRL1320, *L*. *rhamnosus* CRL1332	Healthy human vagina	Vaginal capsule	VM‐restoration	Double‐blind randomized clinical trial of safety. 39 normal or intermediate microbiota women	*Lactobacillus* formulations (1 capsule/day/7 days) tended to decrease in both Nugent score and vaginal leukocyte number, increased cultivable lactobacilli, without adverse effect	129
**Pro‐I:** *L*. *crispatus* EST‐1, EST‐4, EST‐6 **Pro‐II:** *L*. *crispatus* EST‐2, EST‐3, EST‐5	Healthy human vagina	Oral capsule	VM‐restoration	Randomized double‐blind placebo‐controlled crossover trial. 40 reproductive‐age considered healthy women	Pro‐I or Pro‐II (once capsule/day/1‐week followed by 2‐week washout period, continued with second treatment and washout period) were well tolerable, and Pro‐II reduced Nugent score and *Gardnerella vaginalis* counts	165
**F1‐Probiotic**: *L*. *acidophilus* PBS066, *L*. *reuteri* PBS072 **F2‐Probiotic:** *L*. *plantarum* PBS067, *L*. *rhamnosus* PBS070, *Bifidobacterium animalis* subsp. *lactis* PBS075	Gjjg Human feces	Oral capsule+inulin	Recurrent UGTI‐prevention	Randomized placebo‐controlled pilot study. 60 healthy women	F1 and F2 (14 days) colonized vagina and showed *in vitro* anti‐microbial activity against urogenital pathogens	166
*L*. *crispatus* LbV 88, *L*. *gasseri* LbV 150 N, *L*. *jensenii* LbV 116, *L*. *rhamnosus* LbV96	Healthy human vagina	Yoghurt	Adjunct to BV therapy	Double‐blind randomized controlled‐clinical pilot trial. 36 BV women	Yoghurt (twice/day/4 weeks) improved BV‐recovery rate and symptoms, tended to improve VM	167
		Oral capsule (Florium, European‐Patent‐PCT/EP2011/065877)	IM/VM‐reconstitution of herpesvirus‐pregnant	Randomized trial. 60 women with herpes virus infection on the 14‐16th week of pregnancy	Probiotic (2 capsules/day/30 min before meals/‐week) reduced opportunistic pathogens, increased *Lactobacillus* in intestine and vagina, decreased 2–3 times complaints incidence (bloating/discomfort/constipation/mucus‐in‐stool/excessive‐vaginal‐discharge/itching/swelling/ redness‐of‐mucosa), reduced twofold the incidence of placental insufficiency, preeclampsia and fetal distress	168
*L*. *acidophilus*, *L*. *rhamnosus*, *Streptococcus thermophilus*, *L*. *delbrueckii* subsp. *bulgaricus*	UD	Vaginal capsule (Lactagyn^®^)	Subsequent to R‐VVC‐therapy	Randomized trial. 436 VVC women	Probiotic (10 applications/beginning 5th day after azole treatment) reduced clinical complaints, improved microbiological efficacy	169
*L*. *acidophilus*, *L*. *casei*, *L*. *lactis*, *B*. *bifidum*, *B. lactis*	UD	Oral powder (SimFort; Vitafor Nutrientes)	Adjunct to isoflavone to improve menopause genitourinary symptoms	Randomized trial. 60 postmenopausal‐women	Probiotic (one pack) improved isoflavones metabolism after 16 weeks, but failed to yield estrogenic effect on the urogenital tract and relieve vulvovaginal symptoms	170

Abbreviations: AV, aerobic vaginitis; BV, bacterial vaginosis; GBS, Group B *Streptococcus*; GDM, gestational diabetes mellitus; HPV, human papilloma virus; HSV, Herpes simplex virus; IM, intestinal microbiota; PPROM, preterm premature rupture of membranes; TV, trichomoniasis; UD: undetermined; VM, vaginal microbiota; VVC, vulvovaginal candidiasis, R‐VVC, recurrent‐vulvovaginal candidiasis.

Different colors are used to include different women status in the same table. Each color represents a different state: non pregnant (white), pregnant (light gray), and post‐menopausal (dark gray).

^a^Products in the market.

In non‐pregnant, fertile women (Table 1, white panel), probiotic strains were assayed to: evaluate their safety, and to improve the VM of healthy women^129^, ^139^, ^143^, ^151^; restore the abnormal VM^129^, ^140^, ^159^, ^165^; prevent recurrent UGTI^141^, ^166^; as adjunct treatment to BV,^37^, ^160^, ^161^, ^167^ AV^160^ antimicrobial therapy, recurrent‐VVC (R‐VVC),^152^, ^162^ and TV in BV presence^144^; as BV and R‐VVC subsequent treatment^141^, ^162^, ^169^; and HPV clearance.^145^, ^153^ Most of the publications have evaluated commercial, or potential probiotic formulas, including oral and vaginal capsules, vaginal tablets, suppositories or powders, and even yogurt, containing individual or combined *Lactobacillus* strains of urogenital, intestinal, or food origins, as shown in Table 1. *Lactobacillus* strains combined with bovine‐lactoferrin were also assayed.^37^, ^151^, ^152^ Independent of the administration route, the strain/s and doses evaluated, most of the trials reported positive effect on the UGT, such as the absence of adverse effects, VM improvement and restoration, permanence and colonization of probiotic microorganism, and higher cure rates and lower recurrences, mainly in BV, AV, R‐VVC, and TV patients. However, Marcotte et al.^161^ indicated that the probiotic adjunct therapy of BV did not improve cure rates or alleviate recurrence, probably due to treatment failures or to the limited power of the study. Referred to the use of probiotic for HPV clearance, Palma et al.^145^ reported the higher HPV clearance in BV women with metronidazole therapy plus 6 months vaginal *Lactobacillus* implementation, than the one with only 3 months’ use. Ou et al.^153^ published that the application of probiotic strains did not influence genital high risk‐HPV clearance, but decreased the rates of mildly abnormal and unsatisfactory cervical smears.

Probiotic application in pregnant women (Table 1, light gray panel) indicates that most of the clinical trials evaluated oral administration of commercial formulas containing urogenital *Lactobacillus* strains. A positive effect on the modulation of VM, increased beneficial microorganisms, and lower pathogen levels, as Group B *Streptococcus* (GBS) was reported.^150^, ^157^, ^168^ However, oral probiotics taken from early pregnancy showed no effect on vaginal health during mid and end gestation,^155^, ^156^ or 3 weeks doses or until birth in 36‐week‐pregnancy were ineffective in impacting GBS vaginal colonization.^158^ The addition of vaginal probiotics to standard antibiotic treatment on perinatal outcome in preterm premature rupture of membranes pregnancy prolonged the bith gestational age, the latency period length, improving newborn weight and health.^163^ On the other hand, the oral application of probiotic in maternal health during pregnancy, postpartum, and in infant eczema and allergy prevention was evaluated.^146^, ^147^, ^148^, ^149^ *L*. *rhamnosus* HN001 during pregnancy reduced gestational diabetes mellitus (GDM) prevalence, particularly among older women and those with previous GDM,^147^ and the depression and anxiety scores in women in the postpartum period.^148^ However, *L*. *rhamnosus* HN001 did not reduce the infant eczema.^149^ In other way, Anoshina^168^ showed that combined *Lactobacillus* strains to HSV‐women decreased the complaints incidence of bloating, discomfort, constipation, mucus in stool, excessive vaginal discharge, itching, swelling and mucosa redness, reducing the placental insufficiency and preeclampsia as well as fetal distress incidence.

In postmenopausal women, the application of probiotic strains (Table 1, dark gray panel) in intimate care ointment demonstrated the successful colonization of probiotic strains to improve the healthy VM.^164^ But, oral administration of probiotic+isoflavone was not as effective as the hormonal therapy to improve genitourinary menopause symptoms.^170^

In conclusion, several trials have demonstrated that single or combined probiotic or potential probiotic strains, either alone or supplemented to antimicrobial therapy, were effective for BV, AV, R‐VVC, and TV prevention and treatment. However, there is insufficient clinical evidence on the efficacy of probiotics for vaginal health in pregnant, post‐menopausal women, and for the prevention of preterm birth and its complications. Then, more studies with well‐designed randomized controlled trials evaluating larger patient size, and different: length of interventions, dosage and strains of probiotics, and administration route are urgently required.

## 7 HYGIENE PRODUCTS FOR THE PREVENTION OF UGTI

The probiotic administration in hygiene products, as tampons and pads, has emerged as a new possibility to carry beneficial strains and exert the claimed effect. The pharmacological aspects of the probiotic products for feminine hygiene including sanitary towels and tampons, classified in several countries as medical devices, are scarcely described. They can act in the VM restoration, and in some cases, could reduce the risk associated with toxic shock syndrome produced by *Staphylococcus aureus* strains, with higher incidence in women who use tampons.^171^ The availability of everyday feminine hygiene products (e.g., tampons, sanitary napkins, panty liners) containing probiotic microorganisms from vaginal origin is limited in the market and available only in some regions of the world. Table 2 shows the feminine hygiene products containing vaginal probiotic lactobacilli assayed for pH balancing and protection during menstrual bleeding. Most of them were designed with Lacto Naturel, LN^®^ formula, as tampons, sanitary pads, foams, and creams, with patented probiotic strains: *L*. *gasseri* LN40, *L*. *fermentum* LN99, *L*. *casei* ssp. *rhamnosus* LN113, *Pediococcus acidilactici* LN23. These strains were isolated from vagina and incorporated as freeze‐dried cells. In tampons and towels, they are included in the inner part, and once hydrated, they diffuse to the surface in the vaginal cavity. Different trials of Ellen^®^ probiotic tampons are included in the scientific summary, but the scientific‐reviewed publications were not found.^172^ This summary describes the clinical studies performed, indicating that the use of probiotic tampon can lead to vaginal colonization of LN^®^ probiotic bacteria in asymptomatic women. These devices are aimed to prevent a disturbed VM condition, while the LN‐bacteria exert their effect exclusively in the vaginal cavity and on/in the mucus covering the squamous vaginal epithelial cells. The release of probiotic bacteria and the production of lactic acid are the main proposed effects.

**TABLE 2:** Feminine hygiene products containing vaginal probiotic lactobacilli

Microorganisms	Brand name and type	Claimed effect	References
Lacto Naturel, LN^®^: *L. gasseri* LN40, *L. fermentum* LN99, *L. casei* ssp *rhamnosus* LN 113, *Pediococcus acidilactici* LN23	ELLEN^®^ probiotic tampon	VM‐improvement/restoration	172
	Muvagyn^®^ probiotic tampon		
	Florgynal^®^ probiotic tampon‐Saforelle		
	Natura Femina‐Ellen Tampon		
	Ellen^®^ LN intimate cleansing foam and intimate grooming/shaving cream	Vaginal pH‐maintenance	
	Ellen^®^ probiotic intimate topical cream	pH‐maintenance, hydration dry mucous	
	Natura Femina probiotic paste in cotton sanitary towels	VM and pH‐maintenance, reduce discomfort (itching/irritation/discharge/odors), infection or inflammation	173
*L. acidophilus* pure culture	Carin/Oasis/Micci ProBiotic ultra wings‐sanitary napkins	VM‐improvement/restoration	174
	Intimea LACTOPROBIOTIC (probiotics+lactic acid), ultra‐thin‐dairy use, incontinences	VM‐improvement/restoration+lactic acid to prevent infection and vaginal inflammation	175, 176

Abbreviation: VM, vaginal microbiota.

A group of experts from the Polish Gynecological Society evaluated the available bibliography and issued a statement concluding that this product is an innovative solution allowing the vaginal application of strains with beneficial probiotic properties during menstrual bleeding.^177^ Sauperl et al.^173^ have later proposed the incorporation of a probiotic paste with the same LN strains to the surface of sanitary napkins in the design of functionalized sanitary products.

Other sets of products are Carin/Oasis/Micci ProBiotic, containing *L. acidophilus* in the inner area of feminine sanitary napkins, while Intimea LACTOPROBIOTIC feminine pads include *L. acidophilus* on the surface, registered in United States and Europe patents.^175^, ^176^ Dried lactobacilli are added through contact sorption drying carriers in a lipid phase. The isolation source of these strains neither their effect is unknown.

On the other hand, nanotechnology applying the electrospinning technique has recently been proposed to cover different devices for vaginal uses with viable *Lactobacillus* in a single step.^178^, ^179^ The coating system was successfully applied in several products, as stent, soybeans, cellulose paper, with a wide variety of bioactive immobilized in nanofibers through electrospinning.^180^, ^181^, ^182^ Then, this *Lactobacillus* immobilization system offers advantages when compared with lyophilized powders, since modifications of the final product are not evidenced, and subsequent processing for their incorporation in the product design are not required. There are few publications on the application of probiotics immobilized in nanofibers for vaginal application available. Škrlec et al.^183^ immobilized probiotic *L*. *plantarum* ATCC 8014 (unknown origin) in nanofibers prolonging the strain viability. In the case of vaginal strains, Nagy et al.^184^ immobilized *L*. *acidophilus* in polyvinylalcohol and polyvinylpyrrolidone nanofibers, suggesting them for BV therapy, but did not prove their efficacy. The immobilization of a *L*. *rhamnosus* CRL 1332 vaginal strain in polyvinylalcohol nanofibers by electrospinning was successfully, maintaining the required viable cell numbers during 360 days at 4°C and the inhibition to urogenital pathogens.^178^ Up to date, clinical trials to determine the safety, efficacy and effectiveness of immobilized probiotics in these products for UGTI treatment were not published.

## 8 CONCLUSIONS

*Lactobacillus* species are the predominant microorganisms in the healthy human vaginal microbiome, and their inclusion as probiotic for the UGT in medical clinical practice is widely recommended. Evidence of their multiple and potential effects must be demonstrated, mainly referred to the VM reestablishment and to the preventive/therapeutic effect against UGTI. Urogenital probiotics designed with different bacterial strains, doses, treatment schemes, routes, and vehicles of administration were clinically evaluated. The application in the preterm birth area, in the improvement of maternal health pregnancy and postpartum, and prevention of infant eczema‐allergy must be encouraged. A promising area is the probiotic inclusion in daily use‐hygiene products with nanofiber‐immobilized lactobacilli, with interesting application possibilities. The potential benefits of probiotics use on the health of women around the world strongly support the requirement of further studies to complement the current knowledge and to encourage clinical applications of probiotics in the UGT, either as preventive or therapeutic agents.

## DISCLOSURE

The authors declare there is no conflict of interest. Some of the results of the research group were included in a patent presentation (INPI, 2018, N° 20180103893).
